# Load carriage changes tibiofemoral arthrokinematics during ambulatory tasks in recruit-aged women

**DOI:** 10.1038/s41598-024-60187-3

**Published:** 2024-04-25

**Authors:** Camille C. Johnson, Alex C. Dzewaltowski, Dennis E. Dever, Kellen T. Krajewski, Ajinkya Rai, Nizam U. Ahamed, Katelyn F. Allison, Shawn D. Flanagan, Scott M. Graham, Mita Lovalekar, William J. Anderst, Chris Connaboy

**Affiliations:** 1 Department of Sports Medicine and Nutrition, University of Pittsburgh, Pittsburgh, PA USA; 2 Orthopaedic Biodynamics Laboratory, Department of Orthopaedic Surgery, University of Pittsburgh, Pittsburgh, PA USA; 3 Center of Lower Extremity Ambulatory Research, Rosalind Franklin University of Medicine & Science, Chicago, IL USA; 4 School of Applied Sciences, Edinburgh Napier University, Edinburgh, Scotland UK

**Keywords:** Musculoskeletal system, Occupational health

## Abstract

The introduction of women into U.S. military ground close combat roles requires research into sex-specific effects of military training and operational activities. Knee osteoarthritis is prevalent among military service members; its progression has been linked to occupational tasks such as load carriage. Analyzing tibiofemoral arthrokinematics during load carriage is important to understand potentially injurious motion and osteoarthritis progression. The study purpose was to identify effects of load carriage on knee arthrokinematics during walking and running in recruit-aged women. Twelve healthy recruit-aged women walked and ran while unloaded (bodyweight [BW]) and carrying additional + 25%BW and + 45%BW. Using dynamic biplane radiography and subject-specific bone models, tibiofemoral arthrokinematics, subchondral joint space and center of closest contact location between subchondral bone surfaces were analyzed over 0–30% stance (separate one-way repeated measures analysis of variance, load by locomotion). While walking, medial compartment contact location was 5% (~ 1.6 mm) more medial for BW than + 45%BW at foot strike (p = 0.03). While running, medial compartment contact location was 4% (~ 1.3 mm) more lateral during BW than + 25%BW at 30% stance (p = 0.04). Internal rotation was greater at + 45%BW compared to + 25%BW (p < 0.01) at 30% stance. Carried load affects tibiofemoral arthrokinematics in recruit-aged women. Prolonged load carriage could increase the risk of degenerative joint injury in physically active women.

## Introduction

With the U.S. military opening ground close combat roles (i.e., infantry) to women^1^, research into specific demands placed on female recruits during physical training is required to help minimize injury, optimize performance, and ensure combat readiness^2^. Women in the military are believed to be at greater risk of sustaining a lower extremity musculoskeletal injury during load carriage activities due to (i) anatomical differences (i.e., overstriding as a compensation for smaller stature, leading to stress fractures)^3^, (ii) disparities in physical fitness at the onset of basic combat training (BCT)^4–6^, and (iii) biomechanical responses to load carriage^7^. During BCT, female trainees sustain twice as many injuries and have a 2.5× greater risk of significant time-loss injuries than their male counterparts^4^. Female trainees also have an increased risk of sustaining meniscal or ligamentous injuries compared to more senior female military members^8^. The absolute nature of the loads carried and relative differences in morphology between the average male and female warfighter may lead to exacerbated effects of load on individuals who are smaller in stature; proportionally affecting women to a greater extent.

A key concern is that these carried loads may increase internal joint loads and leave female trainees at a higher risk for development of osteoarthritis (OA). Two potential mechanisms of OA initiation and progression are (1) alterations in the location of cartilage loading and (2) short-term loss of cartilage thickness due to repeated loading. Cartilage thickness changes during running^9^ and has been shown to deform following bouts as short as 15 min^10^. Joint loading without sufficient cartilage conditioning or recovery is related to cartilage degeneration, and by extension OA^11–14^. Therefore, repeated bouts of walking or running over long distances with additional carried load could be a potential mechanism for OA onset and contribute to the increased OA risk seen in warfighters across their military service^15^. Additionally, a recent systematic review reported that female military members are more at risk for musculoskeletal injury (MSI) of the lower limbs than their male counterparts^8^, with carrying loads exceeding 30 lb^16^ or 10% of an individual’s bodyweight^17^. Increased knee flexion during load carriage activities have been previously reported^18–20^, suggesting that altered gait strategies resulting from heavy load carriage may play a role in this increased risk of soft tissue injury.

The purpose of this study was to identify the effects of carried loads on women’s knee kinematics (tibiofemoral rotations and translations), compartmental joint space, and location of closest contact between the subchondral bone surfaces during walking and running using biplane radiography. Biplane radiography provides a reliable and accurate method for capturing *in-vivo* bone motion, including subchondral bone distances, during dynamic movements using short-duration x-ray pulses at high repetition rates to image dynamic movements with minimal motion blur (accuracy: < 1.0 mm in translation and < 1.0° in rotation, vs. errors of 8.3–16.9 mm and 3.3–13.1° for marker-based motion capture, with the largest errors occurring in the transverse plane)^21–24^. We hypothesized that increased load carriage would be associated with increased tibiofemoral flexion, decreased joint space, and a more medial and posterior location of the center of closest contact between subchondral bone surfaces during impact and early stance phase of gait.

## Results

**Figure 1:**
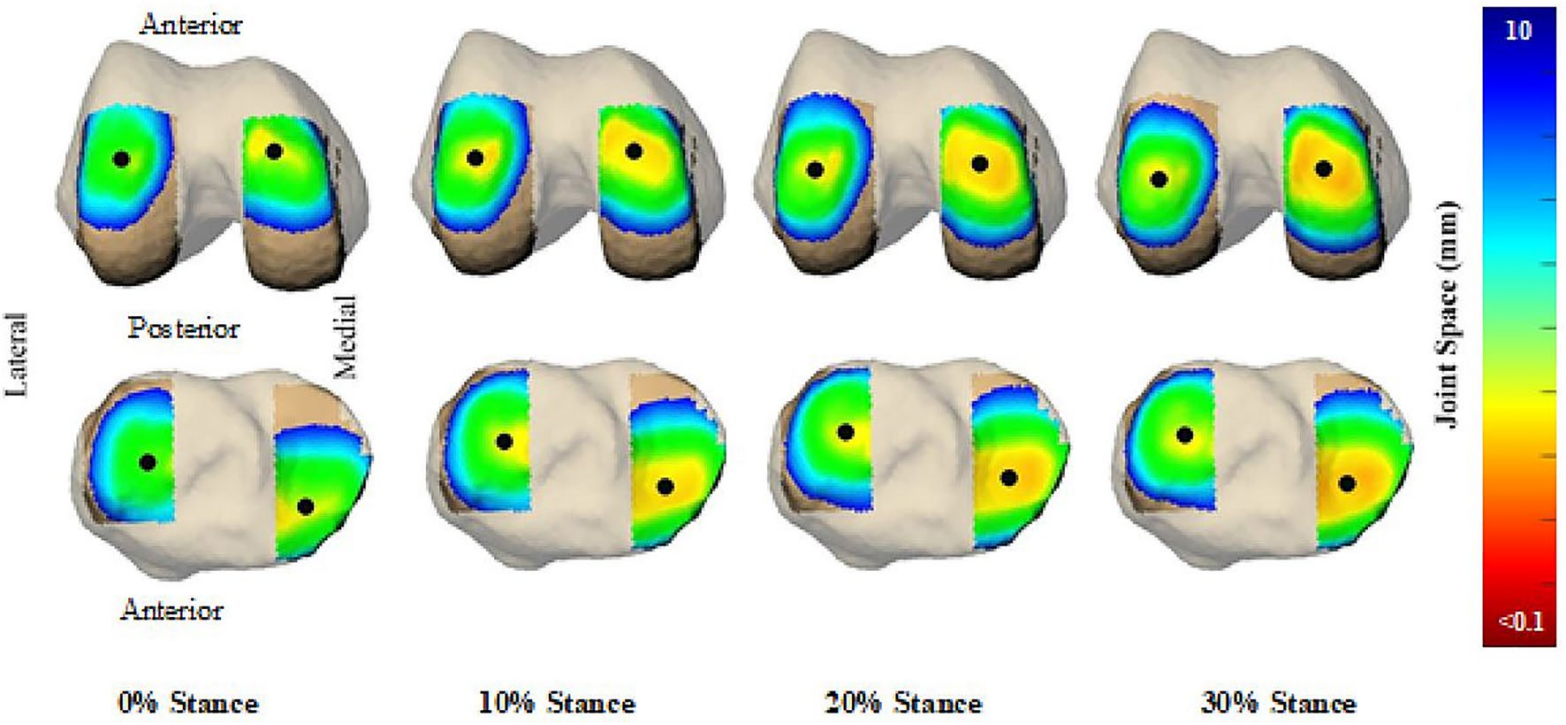
Contact center location for a single participant and average minimum tibiofemoral gap for all participants. Joint space (color map) and contact center location (black ball) between the femur (top) and tibia (bottom) over 0–30% stance phase for a single subject during a walking trial no carried load (BW).

Twelve healthy recruit-aged women participated in this study. Participant demographics (age: 24.5 ± 2.4 years, height: 161.7 ± 5.7 cm, body mass: 58.1 ± 7.8 kg, BMI: 22.2 ± 2.3 kg/m^2^, percent fat mass: 25.9 ± 9.2%, percent fat free mass: 71.0 ± 8.4%) were comparable to previously studied cohorts of female trainees in the initial phases of basic combat training^3,6^. The three load conditions tested were no additional load [bodyweight, or BW], an additional load of 25% of BW [+ 25%BW], and an additional load of 45% of BW [+ 45%BW]. Carried loads were 14.3 ± 2.0 kg for the + 25%BW condition and 25.6 ± 3.5 kg for the + 45%BW condition. An example of joint arthrokinematics (location of the center of closest contact) for a single participant can be viewed in Fig. 1. Average tibial plateau width (medial–lateral axis) was 22.9 ± 1.9 mm for the medial compartment and 22.2 ± 1.4 mm for the lateral compartment. Average tibial plateau depth (anterior–posterior axis) was 42.2 ± 2.2 mm for the medial compartment and 35.2 ± 2.5 mm for the lateral compartment.

### Walking

**Figure 2:**
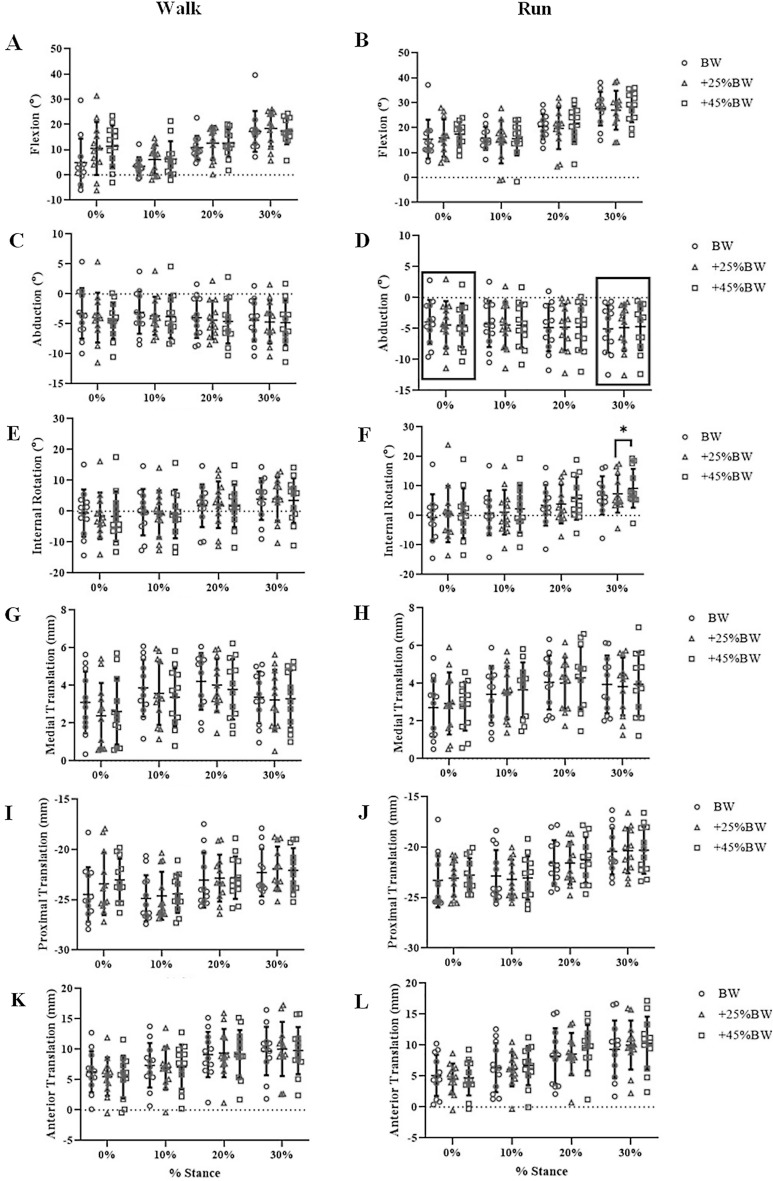
Six degree-of-freedom tibiofemoral kinematics during walking and running. 6-DOF kinematics (flexion(+)-extension(−), abduction(+)-adduction(−), external rotation(+)-internal rotation(−) (°); medial(+)-lateral(−), proximal(+)-distal(−), anterior(+)-posterior(−) translation (mm)) for all subjects (12 per load condition) while walking (Walk) and running (Run) at each time point (0–30% stance at 10% intervals). “*” indicates significant differences between loading conditions (p < 0.05). Box and “*” around time point indicate significant main effect of load (p < 0.05), with no significant pairwise comparisons identified.

**Figure 3:**
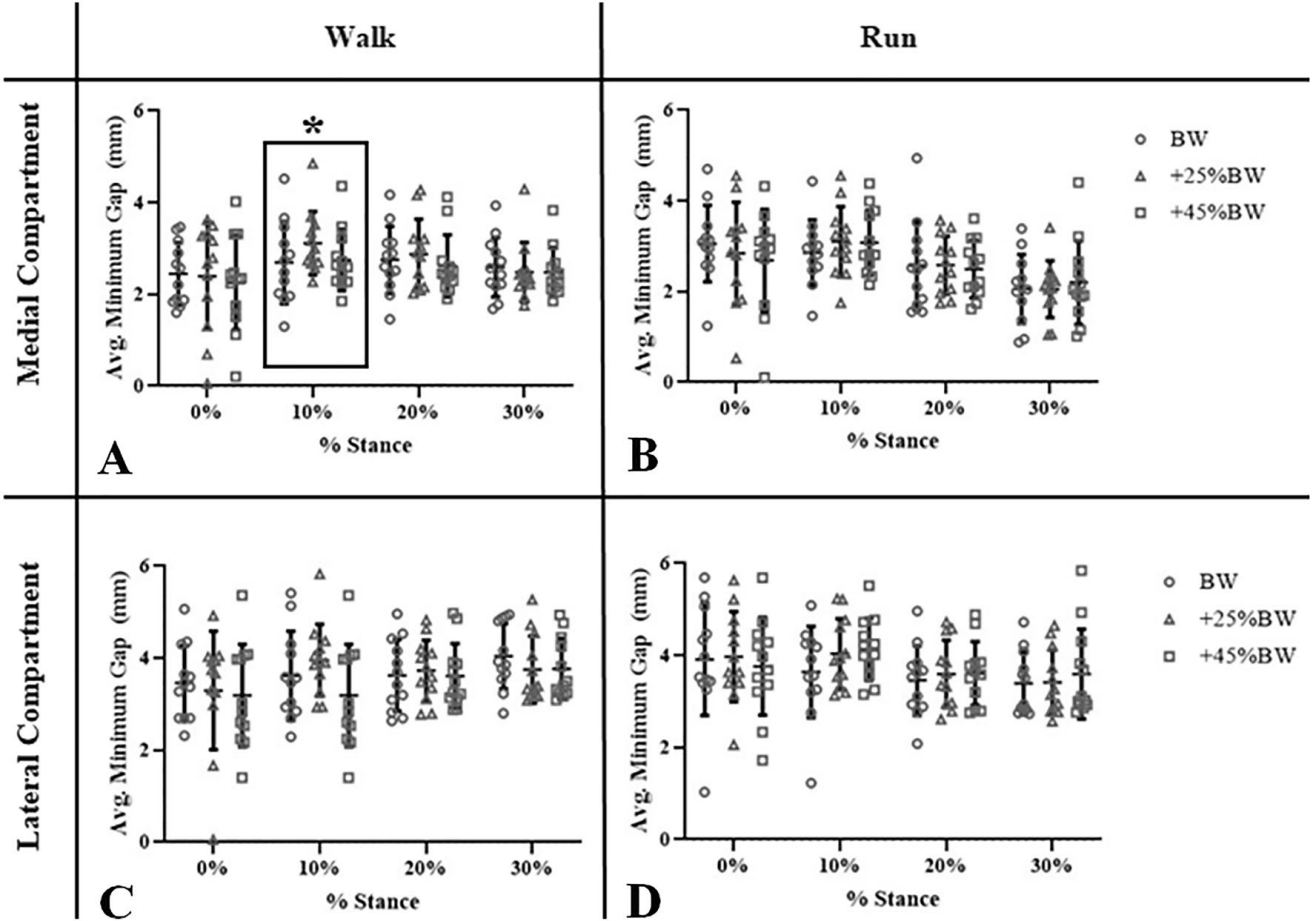
Average minimum gap (mm) in the medial and lateral compartment for all subjects during walking and running for each load condition over 0–30% stance. Box and “*” around time point indicate significant main effect of load (p < 0.05), with no significant pairwise comparisons identified.

Average walking speed for all participants was 1.6 ± 0.2 m/s. No significant differences due to load condition were observed for flexion, abduction, internal rotation, medial translation, proximal translation, or anterior translation during the walking trials (all p > 0.05) (Fig. 2A,C,E,G,I,K). At 10% stance, medial compartment minimum gap was significantly affected by load (Fig. 3A, p = 0.04, η^2^ = 0.26); pairwise comparisons did not reveal significant differences between loading conditions. The medial and lateral contact center location was significantly affected by loading condition along the medial–lateral axis at foot strike (p = 0.01, η^2^ = 0.32 and p = 0.02, η^2^ = 0.28) (Table 1); the contact center in the medial compartment was 5% (approximately 1.6 mm [Table 2]) more medial in the BW condition than the + 45%BW condition at foot strike (p = 0.03), while no significant pairwise comparisons were detected in the lateral compartment contact center location. The medial contact center location was also significantly affected by loading condition along the anterior–posterior axis at 30% stance (p = 0.03, η^2^ = 0.26) (Table 1); no significant pairwise comparisons were detected.

**Table 1:** Average contact center location during walking (normalized to compartment width and depth, expressed as %) along medial–lateral (ML) and anterior–posterior (AP) axes of the medial and lateral tibial plateau.

	% Stance	Walk					
		ML			AP		
		BW	+ 25% BW	+ 45% BW	BW	+ 25% BW	+ 45% BW
Medial compartment	0	32*	32*	38*	68	64	64
	10	34	36	37	68	66	65
	20	42	46	44	60	57	58
	30	39	42	42	56*	52*	54*
Lateral compartment	0	36*	41*	41*	57	54	55
	10	33	36	35	55	54	54
	20	33	33	34	47	45	45
	30	37	37	38	41	38	39

Along the ML tibial plateau axis, 0% indicates medial border while 100% indicates lateral border of either compartment. Similarly, along the AP tibial plateau axis, 0% indicates the posterior border while 100% indicates the anterior border of either compartment. “*” indicates significant effect of load.

**Table 2:** Average absolute difference in contact center location (mm) between loads during walking and running, expressed as mean (SD), along medial–lateral (ML) and anterior–posterior (AP) axes of the medial and lateral tibial plateau.

		% Stance	ML			AP		
			+ 45%BW − + 25%BW	+ 25%BW − BW	+ 45%BW − BW	+ 45%BW − + 25%BW	+ 25%BW − BW	+ 45%BW − BW
Walk	Medial	0	1.7 (1.3)	1.1 (0.9)	1.6 (1.3)	2.1 (1.4)	2.6 (3.1)	2.9 (2.6)
		10	1.4 (1.0)	0.9 (0.9	1.3 (1.1)	1.5 (1.9)	1.5 (1.4)	2.0 (2.9)
		20	1.2 (0.9)	1.5 (0.2)	1.0 (1.0)	1.6 (1.0)	2.0 (2.2)	2.3 (2.8)
		30	1.0 (0.8)	1.2 (1.0)	1.3 (1.1)	1.5 (0.9)	1.8 (2.0)	1.2 (1.4)
	Lateral	0	0.9 (0.7)	1.1 (1.7)	1.3 (1.1)	1.5 (1.1)	1.6 (1.7)	1.9 (2.6)
		10	0.7 (0.6)	0.7 (0.8)	0.9 (0.8)	1.2 (0.9)	1.6 (1.8)	1.8 (2.5)
		20	0.3 (0.3)	0.7 (0.7)	0.7 (0.8)	1.6 (1.1)	2.2 (1.6)	2.0 (1.6)
		30	0.5 (0.6)	0.7 (0.6)	0.8 (0.7)	1.2 (0.9)	2.0 (1.9)	1.8 (1.8)
Run	Medial	0	0.8 (0.5)	1.3 (1.6)	1.5 (1.1)	2.0 (1.4)	2.5 (2.5)	1.8 (2.4)
		10	0.8 (0.6)	1.1 (0.8)	1.3 (1.0)	1.5 (0.7)	1.9 (1.3)	2.0 (1.7)
		20	0.7 (0.5)	0.7 (0.6)	0.9 (0.6)	1.1 (1.4)	2.7 (2.0)	2.8 (2.7)
		30	0.7 (0.6)	0.8 (1.0)	1.1 (1.2)	0.9 (1.3)	1.9 (2.7)	2.1 (2.8)
	Lateral	0	0.5 (0.4)	0.9 (0.9)	0.8 (0.9)	0.7 (0.8)	1.3 (0.9)	1.2 (1.2)
		10	0.6 (0.4)	0.8 (0.5)	0.5 (0.5)	0.9 (1.0)	1.7 (1.6)	1.4 (2.1)
		20	0.6 (0.5)	0.7 (0.6)	0.8 (1.0)	1.5 (1.1)	2.2 (2.0)	2.7 (2.7)
		30	0.3 (0.3)	0.6 (1.0)	0.6 (1.1)	1.3 (0.7)	1.7 (2.3)	1.9 (2.8)

### Running

Average running speed for all participants was 2.0 ± 0.2 m/s. No significant changes in knee flexion or medial–lateral translation, proximal–distal translation, or anterior–posterior translation were observed with increasing load during the running trials (Fig. 2B,H,J,L). At foot strike, abduction was significantly affected by load (Fig. 2D, p = 0.02, η^2^ = 0.30); pairwise comparisons between loading conditions did not reach the level of statistical significance. At 30% stance, abduction and internal rotation were significantly affected by load (Fig. 2D and F, p = 0.04, η^2^ = 0.30; p = 0.01, η^2^ = 0.33, respectively); internal rotation was higher at + 45%BW compared to + 25%BW at 30% stance (p < 0.001) (25% increase in internal rotation with increased load). No significant pairwise comparisons were identified for abduction at 30% stance. No significant effects of loading were observed for medial or lateral compartment minimum gap (Fig. 3B,D). The medial contact center location was significantly affected by loading condition along the medial–lateral axis at 0% and 30% stance (p = 0.02, η^2^ = 0.27; p = 0.03, η^2^ = 0.32) (Table 3); at 30% stance, the contact center was 4% more lateral (approximately 1.3 mm) during BW than during 25%BW, while no significant pairwise comparisons were identified between load conditions at 0% stance (Table 2).

**Table 3:** Average contact center location during running (normalized to compartment width and depth, expressed as %) along medial–lateral (ML) and anterior–posterior (AP) axes of the medial and lateral tibial plateau.

	% Stance	Run					
		ML			AP		
		BW	+ 25% BW	+ 45% BW	BW	+ 25% BW	+ 45% BW
Medial compartment	0	36*	40*	41*	66	64	65
	10	43	43	43	63	63	63
	20	51	50	50	55	54	52
	30	51*	47*	47*	50	48	48
Lateral compartment	0	40	39	39	54	54	53
	10	36	36	35	50	51	49
	20	34	35	34	43	42	38
	30	35	37	37	35	34	31

Along the ML tibial plateau axis, 0% indicates medial border while 100% indicates lateral border of either compartment. Similarly, along the AP tibial plateau axis, 0% indicates the posterior border while 100% indicates the anterior border of either compartment. “*” indicates significant effect of load.

## Discussion

The purpose of this study was to identify the effects of load carriage magnitude on tibiofemoral arthrokinematics, compartmental joint space, and center of closest contact location between subchondral bone surfaces in recruit-aged women during walking and running. The primary finding of the study was that increased load significantly impacted abduction at foot strike and internal rotation as the participants approached mid-stance (30% stance) while running. We also observed an altered subchondral center location of closest contact at both foot strike and 30% stance while walking and running. However, these shifts in contact location did not affect minimum subchondral joint space during either task.

This study found significant changes in the location of the center of closest contact between subchondral bone surfaces due to changes in load carriage magnitude (Tables 1, 3), but it remains unknown if eventual degradation would occur as participants progressed through repeated load carriage tasks over a long period of time. The absolute differences in contact center location between load conditions were small (up to 2.9 mm, Table 2) and generally near the measurement accuracy of the biplane radiography system (0.7 mm in translation^21,22^). The measurement error associated with the biplane radiography system is indiscriminate and affects the variability among measurement outcomes equally in direction. Therefore, while some of the observed changes are small, the interpretations derived from statistical significance is likely unaffected by the magnitude of the change relative to the measurement system’s error. Although these deviations in contact center location due to load are small (less than 3 mm), it has been hypothesized that deviation from normal kinematic patterns is a driving factor of OA progression, with increased risk of cartilage degeneration as loading shifts to less-frequently loaded portions of the cartilage^13,14^. With an average tibial compartment width and depth of approximately 22 mm and 42 mm for this cohort, a 3 mm shift in contact center location corresponds to ~ 7–14% of the total compartmental dimensions. Further, a 3 mm shift in contact center location along the anterior–posterior axis is larger than previously reported side-to-side differences in contact center location following ACL reconstruction^25^, pointing to the potential of increased risk of cartilage degeneration as the area of cartilage loaded during activity changes. Variations in articular cartilage thickness at the knee play an important role in load distribution, with the thickest sections of cartilage aligning to provide the greatest load-bearing capability at or near full knee extension, protecting against impact^14^. Significant decreases in tibial cartilage thickness (~ 7%) have been reported following high-impact activities (i.e., loaded static flexion and single-leg drop jumps)^11^; coupled with increased tibiofemoral contact forces of up to 19.9% when increasing walking speed and carried load^26^, rotational changes during gait may shift cartilage contact points to areas not specifically adapted to these increased loads, increasing the potential risk of cartilage degeneration and OA^13,14^.

Although knee flexion was not significantly affected by load carriage, we did identify significant changes in internal rotation and abduction that may contribute to changes in the location of the center of closest contact between the femoral and tibial subchondral bone surfaces. Knee flexion during the early stance phase serves as a mechanism of shock attenuation while running (through eccentric muscle action)^27^. The lack of significant changes in knee flexion with added load coupled with a significant increase in tibiofemoral abduction and internal rotation indicates that load carriage introduces a compensatory gait pattern that may contribute to shifts in cartilage contact location and cartilage loading patterns. Repeated loading and bone adaptation are two factors that have been linked to the eventual degradation of articular structures such as knee cartilage^12–14^. However, repeated loading is a necessary consequence of physical training. Bone, cartilage, and muscle growth are all dependent upon repeated exposure to stress from physical training^10,28,29^. The stress associated with the repeated loading needs to be a ‘sub-injurious threshold’ in order to facilitate performance improvements. Critically, the alterations in knee joint kinematics from load carriage likely lowers this ‘threshold for injury’ for any individual regardless of fitness status due to the novelty of the joint loading pattern. The alterations to joint kinematics are separate from the greater impact forces due to the added mass and when combined, likely exacerbate the potential for longitudinal or acute injury. This is further compounded when an individual is unconditioned and beginning a demanding physical training regimen, like military personnel entering basic training, a population which is reported to be at high risk for acute injury^4,8,30^. As alternative example, altered knee abduction has also been shown to be a risk factor for ACL rupture in female athletes^31^ and when combined with increased internal rotation, these abnormal kinematic patterns may put increased tension on the soft tissues stabilizing the knee and lead to subsequent injury.

The relative loads used in the present investigation equated to the lower end of the spectrum (~ 20 kg) than what is traditionally utilized in military settings (which can be up to 60 kg regardless of body size, greater than the current study’s + 45%BW condition [25 ± 3.5 kg])^32^. Periodized load carriage training consists of incremental increases in carried loads to introduce and condition the body to loaded exercise. Future work should investigate the effects of periodized load carriage training for its effectiveness in prevention of detrimental changes in lower extremity kinematics and to the subchondral joint space.

While the significant findings reported from this study were characterized by large observed effect sizes (η^2^ = 0.26–0.32) indicative of the reduced possibility of a type 1 error, the large magnitude of between-subject variability observed indicates that future research may benefit from recruiting a larger sample of participants based on factors such as neutral anatomical position, load carriage experience level, or running type (i.e., forefoot vs. rearfoot striking), to better examine the implications of load carriage. Additionally, the current analysis focused on moderately physically active women walking or running in a straight line on level terrain, so the results cannot be extrapolated to individuals at a different level of physical fitness (i.e. sedentary individuals or professional athletes), men, or activities regarding variable terrain and movement types (i.e., overground marching on rough terrain, or rapidly changing direction while running). Similarly, the load considered in this study was idealized in the sense that it was limited to the torso, balanced evenly in the anterior/posterior direction, and was scaled to the participant’s bodyweight to remove the effects of participant body size. Further, the loads studied represent those carried by warfighters during the early portions of basic training; movement strategies may differ in field-based settings where fatigue may introduce additional compensatory movement patterns. Studying heavier loads that approach those used in standard military operations (20–60 kg) could potentially lead to more identifiable differences. Lastly, this study focused on lower intensity walking and running velocities, so additional research into the higher velocity “forced-march” gait pattern (exceeding the individual’s GTV) that is often evident in military marching activities is necessary to provide a more complete picture of the knee joint’s response to military-relevant load carriage.

Overall, the results of this study indicate that load carriage may play a role in the increased incidence of lower extremity musculoskeletal injury in military populations through altered tibiofemoral arthrokinematics during load carriage activities. Changes in subchondral tibiofemoral articulations and decreased compartmental joint space during loaded walking may contribute to cartilage degradation and early OA onset in younger military populations, particularly in healthy, recreationally-fit recruit-aged women. Future research is warranted to aid our understanding of the connection between load carriage magnitude, load carriage exposure, and their long-term consequences related to cartilage health.

## Methods

### Participants

Twelve right leg dominant healthy young women (mean age: 24.5 ± 2.4 years) consented to participate in the study. All participants were informed of the potential risks of the study prior to obtaining written informed consent. The investigation was approved by the University of Pittsburgh’s internal review board and all experimental methods were performed in accordance with all relevant guidelines/regulations including the Declaration of Helsinki. An a priori power analysis indicated that with a moderate effect size (η^2^ = 0.2) and alpha of 0.05, 11 participants were needed to achieve 80% power when using one-way repeated measures analysis of variance (RMANOVA) to identify arthrokinematic differences due to load carriage magnitude while walking and while running, analyzed separately (G*Power 3.1, Heinrich Heine, Universität Dusseldorf, Germany). Participants were moderately physically active (> 30 min of moderate exercise at least 5 days per week) and comfortable carrying loads up to 45% of their bodyweight. Exclusion criteria were any current musculoskeletal injury affecting the back or lower extremities, a pre-existing condition which would be worsened by participating in activities involving load carriage, or a positive urine pregnancy test.

### Procedures

Participants completed two separate sessions, separated by a minimum of 72 h, to assess body composition, familiarize with equipment and collect arthrokinematics data. During the first session, body composition was assessed using dual-energy x-ray absorptiometry (DEXA) (Lunar iDXA, GE Healthcare, Chicago, IL, USA) total body scan and used to assess overall participant demographics. Participants wore a weighted vest (Short plus style vest, MIR, San Jose, CA, USA) with an equally distributed anterior/posterior load and combat boots (Speed 3.0 Jungle RD, 5.11 Tactical, Irvine, CA, USA) for all subsequent data collection procedures. To familiarize participants with using a treadmill wearing a vest and boots, participants walked and ran on a treadmill following a previously described custom variable-velocity protocol to become accustomed to changing velocities while loaded^2,33^.

For the second session, each participant completed six data collection trials. Three load conditions (no additional load [BW], an additional load of 25% of BW [+ 25%BW], and an additional load of 45% of BW [+ 45%BW]) and two velocity conditions (walk and run) were randomized. Gait transition velocity (GTV), or the velocity at which the participant naturally transitioned from a walking gait pattern to a running gait pattern, was determined for each load condition by allowing the participant naturally transition to a run over a constant treadmill acceleration (0.05 m/s^2^)^2,33^. GTV for that load condition was calculated as the average GTV over three trials. Participants then walked or ran on a dual-belt instrumented treadmill (Bertec Corp, Columbus, OH, USA) where walking velocity was set at 10% below GTV and running velocity at 10% above GTV. Ground reaction forces were recorded from each belt of the treadmill at 1000 Hz. Synchronized biplane radiographs (150 images/s for 1.0 s, maximum 90 kV, 160 mA, 1 ms pulse width) of the dominant knee were collected at dominant-limb foot strike through mid-stance after approximately 90 s of walking or running. Participants were given a rest period of several minutes between trials and load conditions to minimize fatigue.

### Data processing

**Figure 4:**
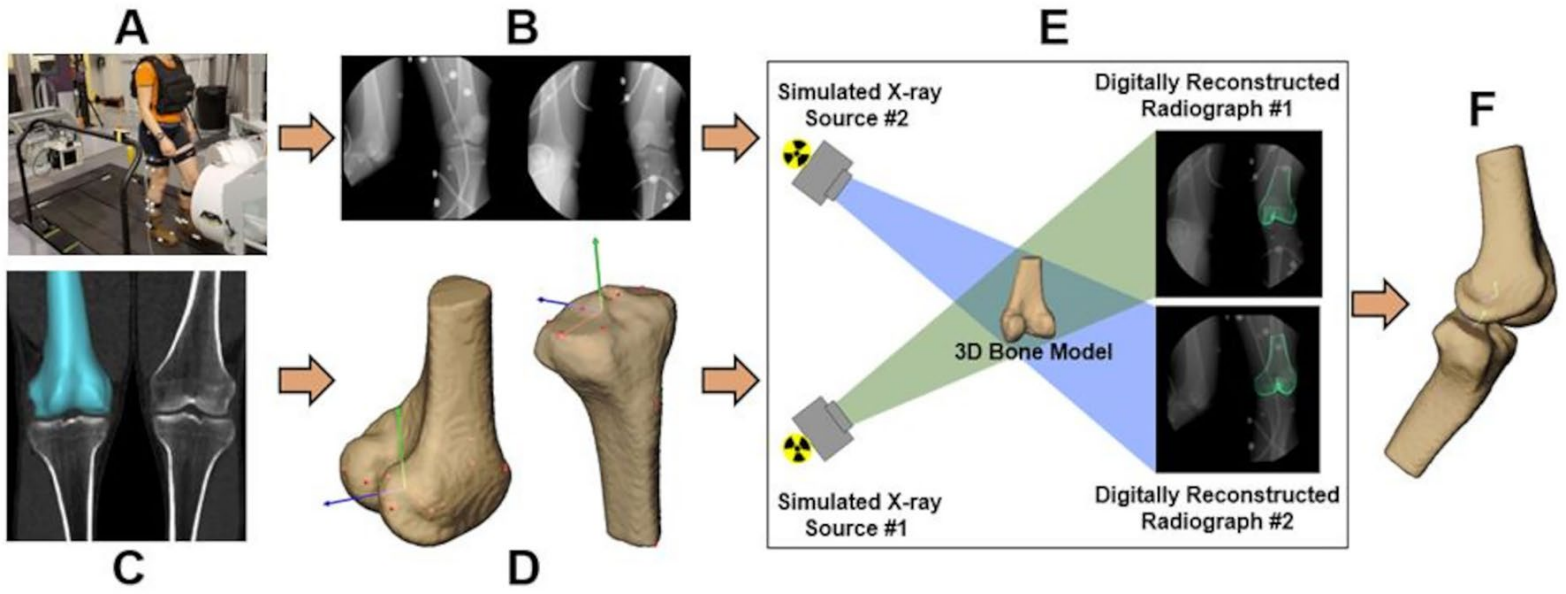
Data processing workflow. The workflow utilized to acquire and analyze biplane radiographic images of dynamic tibiofemoral joint motion. (**A**) Participants walked or ran on an instrumented treadmill while (**B**) synchronized biplane radiographs were collected at 150 images per second. (**C**) CT scans were segmented to create subject-specific 3D bone models with coordinate systems established in (**D**). (**E**) Digitally reconstructed radiographs, created from segmented bone tissue, were matched to the radiographic images in the virtual lab space using a model-based tracking technique. (**F**) Six DOF kinematics of the tibiofemoral joint and subchondral bone distances^23^ were generated for further analysis.

Data were processed using previously described distortion correction, calibration, and registration procedures (Fig. 4)^21^. CT scans (scan dimensions: 0.5 × 0.5 mm in-plane resolution, 1.25 mm slice thickness, and ± 10 cm from the joint line) of the dominant-limb knee were acquired from each participant (Lightspeed 16, General Electric, Boston, MA, USA). Hip and ankle slices were obtained to aid in defining joint coordinate systems for kinematic analysis. Bone tissue was segmented from the CT volume using a combination of commercial software (Mimics, Materialise, Leuven, Belgium) and manual segmentation and was reconstructed into three-dimensional models of the femur and tibia^34^. A previously validated volumetric model-based tracking technique with an *in-vivo* accuracy of 0.7 mm or better in translation and 0.9˚ or better in rotation^21,22^ was utilized to match the digitally reconstructed radiograph to the bone position in each pair of synchronized radiographs (Fig. 4E).

**Figure 5:**
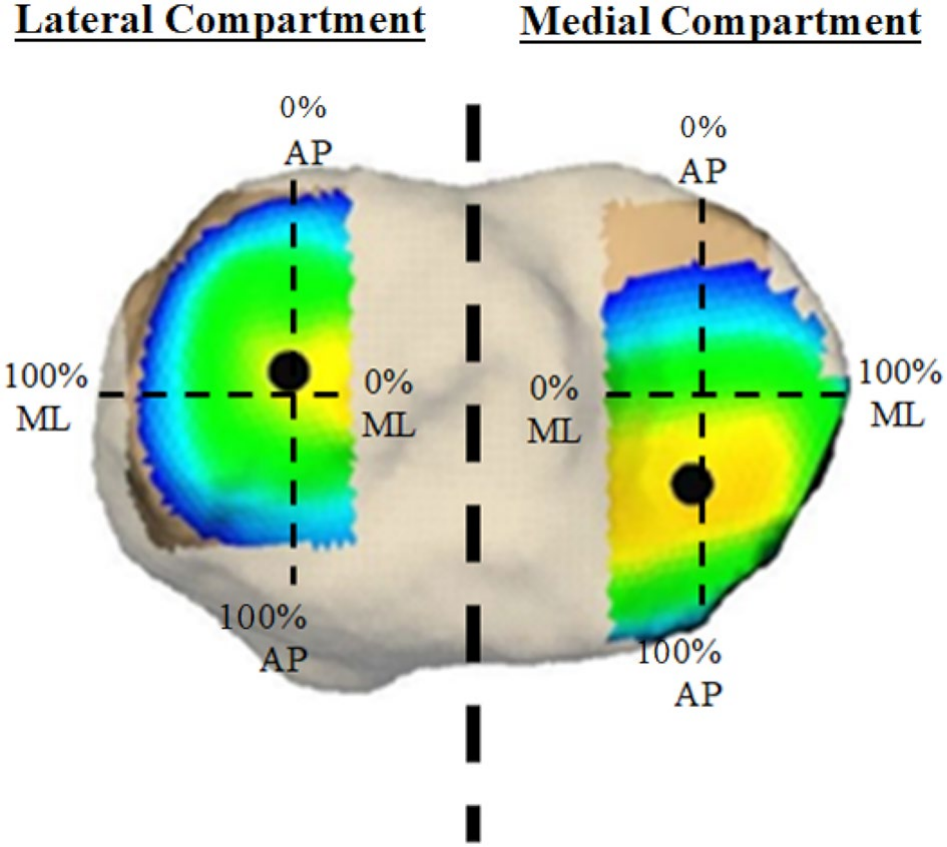
Example of normalized subchondral tibial bone surfaces. Subchondral bone surfaces in the medial and lateral compartment was normalized to each participant’s tibial plateau width and depth using anatomical markers placed on the anterior, posterior, medial, and lateral borders of the tibial bone surface in each compartment and expressed as a percentage. Average contact center location was then expressed along medial–lateral and anterior–posterior axes of the medial and lateral tibial plateau. 0% indicates medial or posterior border of either compartment, while 100% indicates the lateral or anterior border of either compartment.

Anatomic coordinate systems were determined using an automated algorithm based upon articular surface geometry^35^ and single CT slices through the proximal femur and distal tibia^36^. Six-DOF (degrees-of-freedom) kinematics between the femur and tibia were calculated following the International Society of Biomechanics (ISB) recommended standard^37^. Tibiofemoral translations were calculated from the femoral anatomic origin (midpoint between the femoral condylar centers) to the tibial anatomic origin (midpoint between the most medial and lateral points of the tibial plateau) and expressed in the tibial anatomic coordinate system^38^. Foot strike was identified as the instant when the vertical component of the ground reaction force exceeded 50N. The 6-DOF kinematics were interpolated to percent support. Due to the limited imaging volume of the biplane radiography system, the first 30% of the stance phase was captured for all participants. Regions of interest were established on the medial and lateral femoral condyle and the medial and lateral tibial plateau to define subchondral bone surfaces used to calculate minimum joint space and location of closest contact along the anterior–posterior and medial–lateral directions in each compartment^23^. Location of the center of closest contact between the femoral and tibial subchondral bone surfaces in the medial and lateral compartment was normalized to each participant’s tibial plateau width and depth using anatomical markers placed on the anterior, posterior, medial, and lateral borders of the tibial bone surface in each compartment and expressed as a percentage of tibial plateau width and depth (Fig. 5).

### Statistical analysis

Body composition metrics were used for description of the study population’s physical characteristics. Kinematics (dependent variables: tibiofemoral flexion, abduction and internal rotation and anterior, superior, and medial translation) and arthrokinematics (dependent variables: minimum subchondral joint space and location of the center of closest contact between subchondral bone surfaces) were analyzed at 10% increments of stance phase. Descriptive statistics (i.e., mean and standard deviation) were calculated for all variables. Separate one-way repeated measures analysis of variance (RMANOVA) were used to examine the effect of load (3 repeats; BW, + 25%BW and + 45%BW) on tibiofemoral kinematics, joint space, and location of the center of closest contact between subchondral bone surfaces along the anterior–posterior and medial–lateral tibial axis (n = 12 participants for each tested variable) for each locomotion type (walk or run, analyzed separately) at four time points (foot strike [0%], 10%, 20%, and 30% stance phase). One-way RMANOVAs are appropriate because we are interested in the effect of load at each point in the movement cycle. Each phase of stance indicates a different point in the movement and therefore, is self-evident that loading will change as the body moves through a gait cycle. If the assumption of sphericity was violated for any individual RMANOVA (indicated by significant p -value (p ≤ 0.05) for Mauchly’s test of sphericity) then Greenhouse–Geisser adjusted p-values were reported. Post hoc pairwise comparisons were performed using the Bonferroni adjustment as necessary. Alpha level was set to 0.05 for all tests. Eta squared (η^2^) was calculated as a measure of effect size (small effect: 0.01–0.09 moderate effect: 0.09–0.24; large effect: > 0.25)^39^. All calculations were performed using SPSS software Version 26 (IBM Inc.; Armonk, NY, USA).

## Data Availability

Data is available upon reasonable request by contacting the corresponding author at christopher.connaboy@rosalindfranklin.edu.
